# Chronic Kidney Disease With Related Oral Health Problems and Alterations in the Tongue Microbiome Illustrated by a 15-Year-Old Girl: A Case Report

**DOI:** 10.1155/crpe/1018472

**Published:** 2025-02-18

**Authors:** Karolin C. Hoefer, Lutz T. Weber, Nathalie Jazmati, Michael J. Noack, Anna Greta Barbe, Isabelle Graf

**Affiliations:** ^1^Department of Operative Dentistry and Periodontology, Faculty of Medicine and University Hospital of Cologne, University of Cologne, Cologne, Germany; ^2^Department of Pediatric Nephrology, Children's and Adolescents' Hospital, University Hospital of Cologne, Faculty of Medicine, University of Cologne, Cologne, Germany; ^3^Wisplinghoff Laboratories, Cologne, Germany; ^4^Institute for Medical Microbiology, Immunology and Hygiene, University of Cologne, Cologne, Germany; ^5^Department of Orthodontics, Faculty of Medicine and University Hospital of Cologne, University of Cologne, Cologne, Germany

**Keywords:** adolescents, case report, children, chronic kidney disease, dental prevention, oral health

## Abstract

Chronic kidney disease (CKD) is characterised by a gradual loss of kidney function over time and is often linked to oral health issues. This case illustrates typical conditions in young CKD patients based on a 15-year-old girl with CKD stage 4 and methylmalonic aciduria, severe gingivitis and hyperplasia of the gingiva, and a need for orthodontic treatment. Oral hygiene interventions, homecare instructions and deep cleaning under antibiotic shielding were necessary to improve the patient's oral health. The immediate worsening of oral health status after extending the interval between regular prophylaxis appointments highlights the need for intensive prevention in young CKD patients to control oral health disorders. This report aims to demonstrate burden-of-care-reducing oral prophylaxis options for patients suffering from severe CKD with oral manifestations.

**Trial Registration:** German Registry of Clinical Trials: DRKS00010580

**Summary**

  What is new
• Young patients with CKD need regular preventive oral hygiene care. This requires an interdisciplinary approach to reduce the burden of care in patients who already face daily challenges.

## 1. Introduction

Children and adolescents with chronic kidney disease (CKD) exhibit intraoral diseases. Reported effects of CKD on oral health include developmental defects of enamel, tooth staining, craniofacial growth disturbances, stomatitis, gingivitis, halitosis, dry mouth (xerostomia) and distorted taste (dysgeusia) [1–3]; 95.8% suffer from gingival bleeding, 42.5% have calculus, and 16.7% suffer from drug-induced gingival overgrowth (GO) [4]. Preventive dental interventions can influence the oral health of young patients and with adequate compliance have a positive impact. Gingivitis is an inflammatory response to the accumulation of plaque and calculus and is a commonly reported finding in patients with CKD [2]. Inadequate oral hygiene and plaque accumulation is also associated with GO in CKD patients. The Papilla Bleeding Index (PBI) is a measure of gingivitis, which is a consequence of CKD [1, 4]. GO and gum ulcers in the periodontal pockets lead to an additional risk, allowing oral bacteria to invade the systemic circulation [5]. The factors listed had an adverse impact on oral conditions in young patients, illustrating the importance of a needs-based dental prevention programme for young CKD patients [6].

## 2. Case Presentation

**Figure 1:**
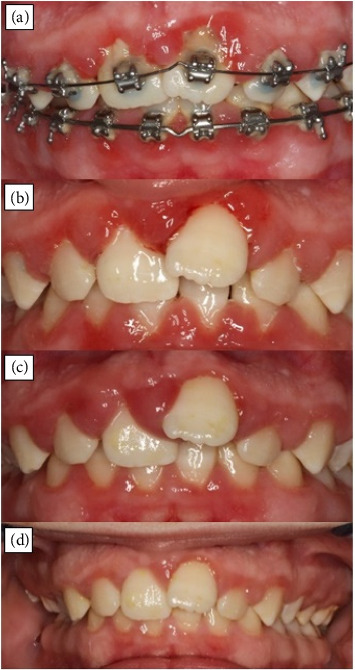
(a) Clinical perspective with multibracket appliance before PMPR, (b) after removal of fixed appliance and an initial PMPR, (c) perspective before removable orthodontic treatment measures with gingival overgrowth and persistent orthodontic treatment need (buccal nonocclusion of second molars on the right and enlarged overjet of 7 mm), (d) following orthodontic treatment with removable appliances (overjet could be reduced and anterior teeth more aligned) and renewed PMPR. Pictures published with the consent of the patient/legal guardian.

The 15-year old girl suffered from CKD stage 4 and methyl malonic aciduria. Secondary diagnoses included hyperparathyroidism and arterial hypertension and severe plaque-induced gingivitis. Upon initial examination (Figure 1(a)), medication comprised sodium citrate, growth hormone, darbepoetin, metoprolol, amlodipine, sevelamer, furosemide, cinacalcet, vitamin D, vitamin B12 and levocarnitine. At the initial appointment in the dental care unit, the girl had a multibracket appliance in the upper and lower jaw. The orthodontist paused the girl's ongoing treatment, despite the need for persistent orthodontic treatment due to the absence of adequate adherence.

The patient attended with aesthetic discomfort and the intention to improve her oral hygiene. Needs-orientated dental interventions were applied to determine whether the severe gingivitis could be reduced solely by oral hygiene interventions. The fixed orthodontic appliance was removed at the dental clinic of the University Hospital in Cologne, Germany, to allow for oral prophylaxis and because the orthodontist had refused to continue the girl's treatment in his private practice. Removable appliances were inserted to meet her persistent orthodontic treatment need; thus, her oral hygiene was impaired as little as possible.

**Figure 2:**
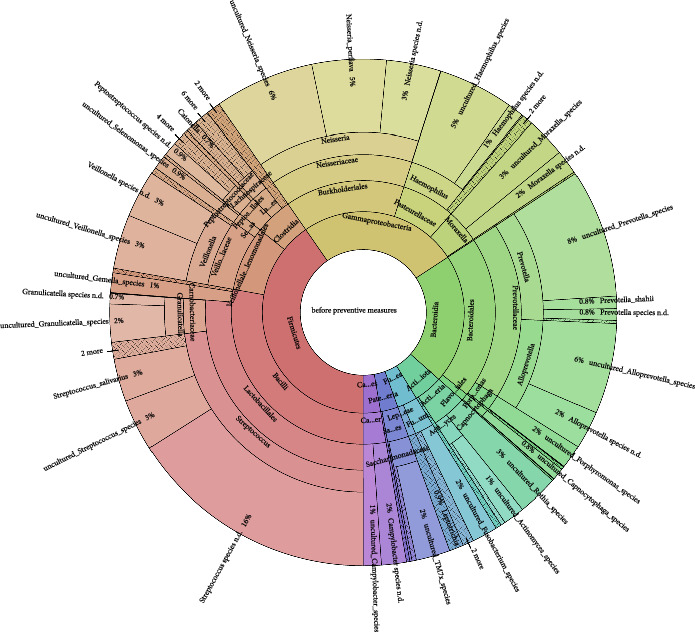
Krona diagram of the tongue microbiome before intensive measures.

**Figure 3:**
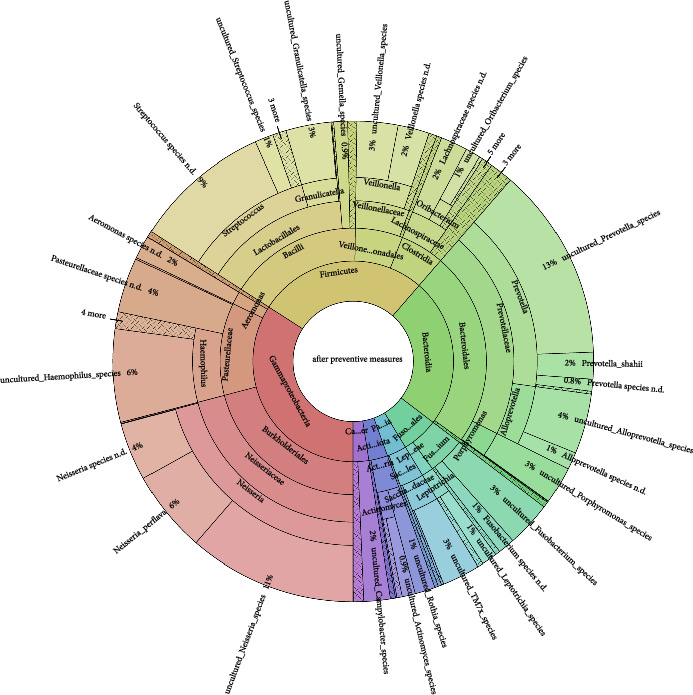
Krona diagram of the tongue microbiome after intensive measures.

The girl received weekly professional mechanical plaque removal (PMPR) over 4 weeks, including oral hygiene instruction. In addition to PMPR, subgingival calculus was removed under an antibiotic shield. Before and after these preventive measures, a swab was taken from the back of the tongue to analyse the resilient tongue microbiome as part of the oral microbiome. The Krona diagram of the initial tongue microbiome showed a typical oral flora, including species associated with gingivitis (e.g. *Fusobacterium* species, *Porphyromonas* species, *Prevotella* species, *Neisseria* species, *Streptococcus* species) (Figure 2). Intensive preventive measures had no substantial impact on the tongue microbiome between different study time points (Figure 3).

When investigations began, the PBI > 0 was measured at 26 measuring points in her oral cavity (Figure 1(a)). Three months after the intensive prophylactic interventions, PBI improved by 80% and PBI > 0 could only be determined at three measurement sites (Figure 1(b)). Besides the PMPR, subgingival calculus was removed from three teeth under antibiotic shielding to decrease local inflammation. Plaque values on the tooth surfaces were reduced to an almost plaque-free oral cavity (Quigley and Hein Index value decreased from 140 to 17). The next appointment took place three months later and showed an increase in local inflammation, with PBI > 0 at 34 measuring points without renewed hygiene instructions. However, intraoral oral hygiene deteriorated once more because of the girl's noncompliance.

## 3. Discussion

We aimed to illustrate whether gingivitis with GO can be treated in a burden-of-care-reducing manner, despite CKD and the use of amlodipine medication known to induce GO. Suffering from any chronic disease is a major challenge for patients and their families and can result in reduced adherence to dental care. Young CKD patients in particular often suffer from multiple symptoms due to their illness, and are not able to differentiate or specify one particular healthcare problem such as GO. Individuals with gingivitis and GO are often burdened by psychosocial impairments and subsequent low oral health-related quality of life.

Although our patient regularly visited her dentist every 6 months, this case highlights that oral care of young CKD patients requires more needs-based dental care than that provided by statutory health insurance. Initial removal of the fixed orthodontic appliance in our patient enabled adequate oral hygiene (Figure 1). Over the following four sessions, PMPR was performed and practised with adapted instructions to implement oral hygiene at home. Due to this intensive interdisciplinary treatment, the formation of soft coatings was minimised and local inflammation and GO were reduced. The enlarged overjet was reduced throughout the course of removable appliance treatment, and upper teeth alignment improved. Without such realignment, adequate oral hygiene would have been persistently impaired, especially combined with GO (Figure 1).

Despite intensive PMPR, dental preventive measures reduced local signs of inflammation and decreasing amounts of plaque, the girl showed no changes in the stable tongue microbiome. This is consistent with a recent publication showing that experimentally induced gingivitis led to significant changes in the abundance of several genera and species in the subgingival and supragingival plaque, while the tongue microbiome showed no changes and was stable throughout the intervention [7]. The tongue microbiome could be the reservoir for gingivitis flora and seems to be resilient [8].

The clinical improvement in gingivitis confirms that targeted preventive measures led to a reduction in clinical symptoms and indices (i.e., PBI). Moreover, clinical benefits were observed that can only be maintained by regular preventive measures and hygiene instructions to ensure the absence of symptoms. In the event of oral health problems that may be related to the patient's medication (i.e., GO after amlodipine in our case), a change in medication should be strongly considered (not an option in our patient).

A typical interdisciplinary team approach for young CKD patients could be as follows: initial screening by the nephrologist for gingivitis with subsequent referral to the dental team for a needs-based oral prevention and treatment programme, then PMPR followed by chlorhexidine and fluoride application, with periodontal surgery if necessary.

The presented patient has been part of a randomized controlled trial [8] about oral health in young CKD patients.

## 4. Conclusion

The establishment and maintenance of oral health in young CKD patients can be achieved through interdisciplinary teamwork. Dental prophylaxis sessions and orthodontic care, together with patient motivation and subsequent adherence and clinical supervision of paediatric nephrologists, were key to improving the oral health of our CKD patient. Due to the already high burden of care in these patients, it is necessary for all professions to provide patient-centered and evidence-based care.

## Data Availability

The datasets generated during and/or analysed during the current study are available from the corresponding author on reasonable request.

## Supporting Information

CARE checklist.
