# Supplementary material for: Chronic Kidney Disease With Related Oral Health Problems and Alterations in the Tongue Microbiome Illustrated by a 15-Year-Old Girl: A Case Report
Source: Case Rep Pediatr. 2025 Feb 18;2025:1018472. doi: 10.1155/crpe/1018472 (PMC11858826; doi:10.1155/crpe/1018472)
Supplement: Supporting Information — Additional supporting information can be found online in the Supporting Information section. [file 1018472.f1.docx]

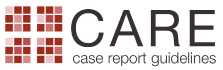

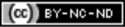

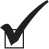


CARE Checklist of information to include when writing a case report

The diagnosis or intervention of primary focus followed by the words “case report” **1-2**

| Topic | Item |
| --- | --- |
| Title | 1 |
| Key Words | 2 |
| Abstract | 3a |
| (no references) | 3b |
|  | 3c |
|  | 3d |
| Introduction | 4 |
| Patient Information | 5a |
|  | 5b |
|  | 5c |
|  | 5d |
| Clinical Findings | 6 |
| Timeline | 7 |
| Diagnostic | 8a |
| Assessment | 8b |
|  | 8c |
|  | 8d |
| Therapeutic | 9a |
| Intervention | 9b |
|  | 9c |
| Follow-up and | 10a |
| Outcomes | 10b |
|  | 10c |
|  | 10d |
| Discussion | 11a |
|  | 11b |
|  | 11c |
|  | 11d |
| Patient Perspective | 12 |
| Informed Consent | 13 |

**Checklist item description**

**Reported on Line**

2 to 5 key words that identify diagnoses or interventions in this case report, including "case report" **22**

Introduction: What is unique about this case and what does it add to the scientific literature?  **24-25**

Main symptoms and/or important clinical findings **14-21**

The main diagnoses, therapeutic interventions, and outcomes **14-21**

Conclusion—What is the main “take-away” lesson(s) from this case? **118-121**

One or two paragraphs summarizing why this case is unique (may include references) 108-116, 102-107

De-identified patient specific information **60-65**

Primary concerns and symptoms of the patient **66-71**

Medical, family, and psycho-social history including relevant genetic information **-**

Relevant past interventions with outcomes **-**

Describe significant physical examination (PE) and important clinical findings **79-86**

Historical and current information from this episode of care organized as a timeline **68-71**

Diagnostic testing (such as PE, laboratory testing, imaging, surveys) **73-75**

Diagnostic challenges (such as access to testing, financial, or cultural)  **-**

Diagnosis (including other diagnoses considered) **16-17, 148-151**

Prognosis (such as staging in oncology) where applicable **-**

Types of therapeutic intervention (such as pharmacologic, surgical, preventive, self-care) **72-72,** **95-101**

Administration of therapeutic intervention (such as dosage, strength, duration) **72-73**

Changes in therapeutic intervention (with rationale  **79-86**

Clinician and patient-assessed outcomes (if available) **108-112**

Important follow-up diagnostic and other test results **113-116**

Intervention adherence and tolerability (How was this assessed?) **89-93**

Adverse and unanticipated events **94-95**

A scientific discussion of the strengths AND limitations associated with this case report **108-112**

Discussion of the relevant medical literature with references 48-58

The scientific rationale for any conclusions (including assessment of possible causes) **88-89**

The primary “take-away” lessons of this case report (without references) in a one paragraph conclusion **118-122**

The patient should share their perspective in one to two paragraphs on the treatment(s) they received **-**

Did the patient give informed consent? Please provide if requested **The parents & the patient**

Yes x No □
